# The Evils of Hot Bread

**Published:** 1883-12

**Authors:** 


### THE EVILS OF HOT BREAD.

There is no law in this country to prevent the consumption of hot bread but the law of common sense, and unfortunately that is a dead letter as a governing principle in the lives of a great many people. That hot bread in nine cases out of ten will produce dyspepsia is no newly-discovered fact, and especially is this terrible result sure to follow persistent indulgence on the part of those whose pursuits are quiet, in-doors and sedentary. And yet the reformers, or those who call themselves such—the men and women who work themselves into a white heat over the sale of a glass of cider—will go on year after year, not only making no outcry against this pernicious indulgence, but actually filling themselves up day by day with the hot and poisonous gases of the oven. This servant of the housewife can be made as terrible a stomach-destroyer as the distillery, and the sworn foes of the latter are apt to be its best patrons. Dyspepsia paints the nose and sours the temper as surely as dram drinking, and many sufferers from the former, though by their own willful acts, inveigh the most loudly against the latter. A well-defined case of jim-jams is the climax to a course of intemperance and warns the victim that his alternative is death or immediate reformation. But the dyspepsia that hot bread, mince pie and kindred abominations cause has no sudden warnings. The man who uses them goes on making both himself and those around him wretched, and refuses to acknowledge that he is a sinner above those whose lighter faults he fiercely condemns.—*American Miller.*

